# A novel bimanual endoluminal robotic platform compatible with standard gastroscopes for endoscopic submucosal dissection: preclinical feasibility and ergonomic evaluation

**DOI:** 10.1007/s00464-026-12972-6

**Published:** 2026-07-01

**Authors:** Yung Lee, Ji Seok Park, Stephen Firkins, Sera Satoi, Vikash Fnu, Kyungran Justina Cho, Amit Bhatt

**Affiliations:** 1 Digestive Disease Institute, Cleveland Clinic, 9500 Euclid Ave A30, Cleveland, OH 44195 USA; 2 University Hospitals Cleveland Medical Center, Case Western Reserve University, Cleveland, OH USA

**Keywords:** Endoscopic submucosal dissection, Endorobotics, Robot-assisted endoscopy, Ergonomics, Operator work load, Endoluminal robotics

## Abstract

**Background and aims:**

Endoscopic submucosal dissection (ESD) enables en bloc resection of early gastrointestinal neoplasia but remains technically demanding because of limited traction, lack of triangulation, and high operator workload, contributing to a steep learning curve. We evaluated a novel endoluminal robotic platform designed to provide surgical-like bimanual triangulation, stable traction/counter-traction, and improved ergonomics while preserving compatibility with standard gastroscopes.

**Methods:**

The robotic system (Intilume System, Agilis Robotics, Hong Kong, SAR) uses an external positioning cart to drive two 3.5-mm flexible robotic instruments mounted onto a native gastroscope via a cap-and-sheath interface and controlled by compact pen-style motion-tracking controllers enabling seated operation. Available instruments included a bipolar T-knife and a tissue grasper. In a randomized crossover ex vivo porcine stomach study, two gastroenterology fellows without prior ESD experience and two interventional endoscopy fellows with early experience each performed four ESDs (two robotic, two conventional), for a total of 16 procedures. Outcomes included en bloc resection, procedure time, specimen surface area, dissection speed, tissue injury, and operator workload assessed using NASA-TLX, with exploratory OSATS and GEARS evaluations.

**Results:**

All procedures were completed (8 robotic, 8 conventional). Robotic ESD achieved 100% en bloc resection versus 75% with conventional ESD (*p* = 0.47), was significantly faster (14.1 ± 4.3 vs 21.6 ± 7.6 min; *p* = 0.028), and demonstrated higher dissection speed (36.5 ± 23.4 vs 16.3 ± 10.1 mm^2^/min; *p* = 0.05). No muscular injuries occurred with robotic ESD compared with 5/8 conventional cases; no full-thickness injuries occurred. Operator workload was markedly lower with robotic ESD (NASA-TLX 34.7 ± 24.1 vs 75.0 ± 15.4; *p* = 0.002).

**Conclusion:**

In a randomized ex vivo model, a bimanual endoluminal robotic platform compatible with standard endoscopes demonstrated promising improvements in ESD efficiency, tissue control, and operator ergonomics compared with conventional ESD. These preliminary findings support further development and progression to first-in-human feasibility evaluation, with potential to facilitate ESD skill acquisition pending clinical validation.

**Graphical abstract:**

**Figure:**
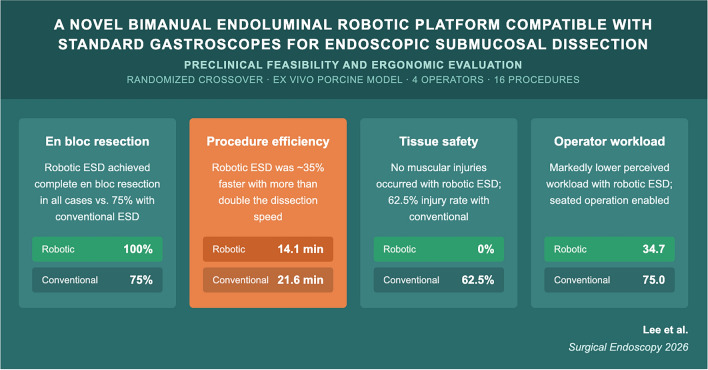

**Supplementary Information:**

The online version contains supplementary material available at 10.1007/s00464-026-12972-6.

Endoscopic submucosal dissection (ESD) is the preferred treatment for select early gastrointestinal neoplasms, offering higher en bloc resection rates and curative outcomes compared with piecemeal resection [1]. Despite these advantages, adoption of ESD in Western practice remains limited, largely due to the procedure’s technical difficulty and steep learning curve [2]. A major challenge lies in the constraints of conventional gastroscopes, which provide only one working instrument. This limits traction and triangulation, effectively leaving the operator with “one hand” inside the lumen, making tissue stabilization and counter-traction difficult [3, 4]. Numerous traction techniques—such as clip-and-suture methods, pulley systems, and dual-channel—have been developed to address this limitation [5, 6]. Although these strategies can improve efficiency, they are often cumbersome and fail to replicate the intuitive bimanual coordination routinely available in surgical practice.

Endoluminal robotic platforms have emerged as a strategy to introduce surgical-style triangulation and enhanced dexterity to flexible endoscopy [7, 8]. Preclinical and early clinical studies have demonstrated that robotic assistance enables safe and efficient ESD, while improving en bloc resection rates and reducing operator workload compared with conventional techniques [6, 8–13]. Collectively, these findings suggest that robotic systems may meaningfully reduce the technical barriers associated with ESD. However, many existing robotic platforms rely on proprietary endoscopes or bulky system architectures that limit scalability and routine clinical adoption. A potential solution would augment a standard endoscope with effective bimanual instrumentation while preserving a familiar and ergonomic workflow for the endoscopist.

In this context, we evaluated a novel endoluminal robotic platform designed to mount onto a conventional gastroscope and provide true bimanual articulation and counter-traction during ESD. The system incorporates two robotic instruments, a bipolar electrocautery knife and a grasper, which are controlled via a compact motion-tracked pen controller, allowing the operator to work in a seated position to help with precision and ergonomics. This study evaluated whether such a robotic platform could enable faster and safer ESD by novice and intermediate endoscopists. A preclinical feasibility comparison of robotic-assisted and conventional ESD was performed in an ex vivo model, with the goal of translating surgical-style triangulation into flexible endoscopy to facilitate broader clinical adoption.

## Methods

### Robotic platform architecture

**Fig. 1:**
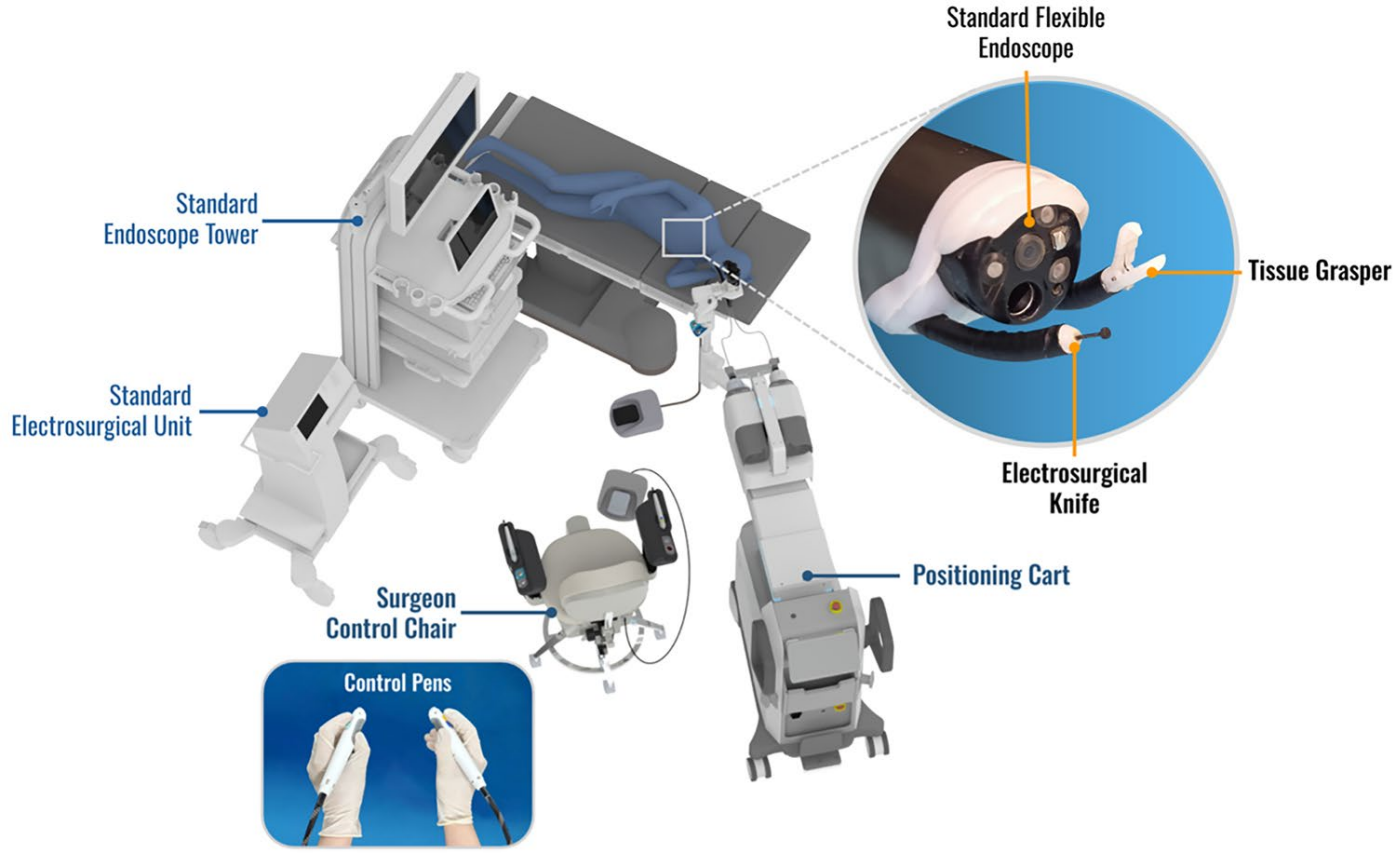
Intraoperative room configuration with the novel endoluminal robotic system

This endoluminal ESD robotic platform (Intilume System, Agilis Robotics, Hong Kong) consists of two main components—a portable positioning cart (external drive unit) and a surgeon console. The positioning cart attaches externally to a standard gastroscope and drives two flexible robotic instruments that mount via a specialized cap-and-sheath on the distal tip of the endoscope. Figure 1 illustrates the system setup, with the portable positioning cart affixed to the endoscope and the slim disposable robotic arms extending over the scope within the operative field. The endoscope itself remains fully functional independently of the robot once it is docked. The endoscope and robotic arms themselves can be disengaged and revert to conventional ESD without exchanging or fully withdrawing the scope. The operator’s console is a small unit housing two precision pen-style motion-tracking controllers, which track the operator’s hand movements to intuitively manipulate the robotic instruments in real time. This pen-based control scheme requires minimal space and allows the endoscopist to operate while seated, in a neutral ergonomic posture. The system’s two 3.5 mm diameter (1.5 m length) robotic instruments, a bipolar “T-knife” electrocautery dissector and a tissue grasper, are inserted through the sheath alongside the endoscope. The entire device goes through an overtube. During use, one instrument (grasper) can provide traction on the target tissue while the other (knife) performs dissection, thereby achieving bimanual coordination and tissue triangulation akin to a surgical operation. The bipolar cautery capability of the T-knife allows both cutting and coagulation during submucosal dissection.

### Study design

We conducted a randomized crossover trial in an ex vivo setting to compare performance of robotic-assisted vs. conventional ESD. Isolated porcine stomachs were used to simulate ESD of gastric lesions. This randomized crossover design was employed to distribute the anticipated rapid skill acquisition of the early learning curve equally across both study arms, thereby mitigating the risk that the robotic group’s performance was merely a result of sequential practice. Two operators performed robotic ESD first (cases 1–2) followed by conventional ESD (cases 3–4), and two performed the reverse order. Allocation was not concealed, as the assigned technique was disclosed to each operator at the beginning of each session prior to scope insertion. On each stomach tissue, an endoscopist estimated a circular lesion of around 30 mm in diameter and marked it on the mucosa. Lesion locations were standardized to the proximal gastric body, as distal gastric mucosa in the ex vivo porcine model tends to be thicker and less representative of typical clinical ESD conditions. Each stomach was utilized until no suitable mucosal surface remained, at which point it was replaced with a new specimen to maintain consistent tissue quality across procedures. A submucosal lift was then created by injecting a viscous dyed solution (Micro-Tech BlueBoost^™^ Submucosal Injectable Solution, Michigan, USA) beneath the lesion. Two gastroenterology fellows with no prior ESD experience (“novice” endoscopists defined by no ESD experience) and two advanced endoscopy fellows with early ESD experience (> 10 cases) during their training participated as operators. Prior to study commencement, all operators received a standardized orientation consisting of a brief demonstration and hands-on benchtop familiarization with the robotic platform. No formal run-in ESD cases were performed ex vivo and no cases were excluded from analysis. Each operator performed four ESD procedures on separate lesions: two using the robotic platform and two using conventional ESD, for a total of 16 ESDs (8 robotic versus 8 conventional). The order of robotic versus conventional ESD for each participant was randomized to mitigate any learning-order effects.

For robotic ESD, a GIF-H190 Olympus gastroscope (Olympus, Tokyo, Japan) was fitted with the robotic sheath and instruments as described above. The operator controlled the robotic arms via the pen controllers at the console while simultaneously advancing or maneuvering the endoscope as needed. For conventional ESD, the same gastroscope was used without the robotic attachment but with a distal plastic cap (Steris, OH, USA) on the tip to facilitate tissue retraction by cap traction. A standard ESD technique was employed: a circumferential mucosal incision and submucosal dissection were performed using an Olympus Dual Knife (KD-650U 2 mm Olympus, Tokyo, Japan) inserted through the endoscope’s working channel. Electrosurgical settings were kept constant in the conventional ESD arm, with the ERBE VIO3 electrosurgery unit (Tubingen, Germany) with identical power settings for cut (EndoCut Q, effect 3) and coagulation (Forced Coag, effect 2, maximum watts 50) in order to standardize the energy delivery across all cases. Conversely, the robotic-assisted arm utilized the energy setting of Bipolar cut + mode at Effect 6 on the ERBEVIO300D electrosurgery unit. Importantly, the operators were not blinded to the technique, given the nature of the intervention; however, all procedures were done on similar tissue models.

### Outcomes and evaluation

We assessed multiple outcome measures to compare the efficacy and safety between robotic and conventional ESD. The primary outcome was the rate of *en bloc* resection, defined as complete resection of the target lesion in one piece with no residual tissue within the given time of 30 min. Cases not completed within 30 min were recorded as primary outcome failures regardless of partial resections achieved. Key secondary outcomes included the following: (1) procedure time (in minutes), measured from the start of the mucosal incision to completion of specimen excision; (2) dissection speed, calculated as resected specimen area (mm^2^) divided by dissection time (minutes), as a metric of efficiency; (3) specimen size (surface area, mm^2^) was measured by photographing the pinned specimen and calculating surface area using ImageJ software by an unblinded assessor (Maryland, USA); and (4) tissue injury incidence, specifically any muscularis propria injuries (Grade 0, no exposure of muscularis propria; Grade 1, muscularis propria exposure; Grade 2, torn muscularis propria; and Grade 3, perforation) or full-thickness perforations, as noted by inspection of the resection bed [14]. These served as surrogates for safety, since unintended muscle or full-thickness injuries reflect technical errors.

Operator workload and strain, quantified by the NASA Task Load Index (NASA-TLX) survey, were administered immediately after each procedure. This well-validated instrument provides a composite workload score (0–100 scale) based on mental, physical, temporal demand, effort, performance, and frustration ratings [15, 16]. A higher NASA-TLX score indicates greater perceived workload/stress. Additionally, qualitative feedback on ergonomics and instrument control was collected from two independent practicing advanced endoscopy attendings with ESD expertise who observed the entire procedure to reduce individual observer bias. Two structured assessment tools were used: the Objective Structured Assessment of Technical Skill (OSATS)—adapted for ESD to rate aspects like tissue handling, time/motion, and use of assistance—and the Global Evaluative Assessment of Robotic Skills (GEARS)—to gauge proficiency in using the robotic system (assessing bimanual dexterity, efficiency, etc.) [17, 18]. The expert observers were not blinded to the technique, operator identity, or case sequence. These were applied for exploratory analysis of how the robotic platform might affect user skill performance and learning over successive cases.

All statistical analyses were performed using Stata statistical software (StataCorp, version 17). Given the small sample per group (8 procedures each), we primarily reported descriptive outcomes and used independent samples *t*-tests or Fisher’s exact tests where appropriate to compare groups. A two-sided *P* < 0.05 was considered statistically significant. Due to the limited sample size and only two procedures per modality per operator, formal mixed-effects modeling was not performed because of model instability concerns. Given the exploratory nature of this pilot study, results are presented descriptively with effect sizes; *p*-values are provided for reference only and should be interpreted with caution given the small sample size. As this study utilized only excised animal tissue and did not involve live animals or human subjects, it was exempt from institutional review. Institutional Animal Care and Use Committee (IACUC) approval was not required, as confirmed by our institution’s research oversight (Cleveland Clinic, OH, USA).

## Results

All 16 ESD procedures (8 robotic-assisted and 8 conventional) were completed successfully by four participants. None of the robotic cases had to be aborted. The performance outcomes for robotic vs. conventional ESD are summarized in Table 1.

**Table 1:** Comparative ESD performance outcomes: robotic vs. conventional

	Robotic (*n* = 8)	Conventional (*n* = 8)	*P*
En-bloc resection (within 30-min time threshold)	8 (100%)	6 (75.0%)	0.47
Procedure time in minutes	14.1 (4.3)	21.6 (7.6)	0.028
Muscular injury	0 (0%)	5 (62.5%)	0.20
Full thickness injury	0 (0%)	0 (0%)	–
Specimen surface area, mm^2^	491.5.(240.9)	288.2 (150.1)	0.07
Specimen area (% relative to ideal target surface area)	69.5 (34.1)	40.8 (21.2)	0.08
Dissection speed mm^2^/min	36.5 (23.4)	16.3 (10.1)	0.05
NASA-TLX score	34.7 (24.1)	75.0 (15.4)	0.002

Values are mean ± SD for continuous measures and n/*N* (%) for categorical measures. Ideal target size was a 30 mm diameter specimen.

*P* values by *t*-test or Fisher’s exact test as appropriate

### Efficacy of resection

Robotic-assisted ESD achieved an en bloc resection in all 8/8 cases (100%), whereas conventional ESD achieved en bloc resection in 6 out of 8 cases (75%). Although this 25-point difference favored the robot, it did not reach statistical significance given the sample size (*P* = 0.47). Notably, the two failures of en bloc resection in the conventional group were due to the operators aborting the procedure after encountering difficulty maintaining the dissection plane; in both instances, large portions of the lesion could not be resected en bloc within a 30-min time limit, whereas the same operators were able to resect similar lesions fully with the robot. With an intended lesion diameter of 30 mm (ideal surface area ≈707 mm^2^), robotic-assisted ESD achieved specimens closer to the target size than conventional ESD. Among completed resections, the mean specimen surface area was 491.5 ± 240.9 mm^2^ in the robotic group (*n* = 8) compared with 288.2 ± 150.1 mm^2^ in the conventional group (*n* = 6; *P* = 0.077). This represented mean shortfalls from the target lesion size of 215.5 mm^2^ for robotic cases and 418.8 mm^2^ for conventional cases, respectively. Although this difference did not reach statistical significance, the numeric trend favored robotic-assisted ESD, suggesting greater ability to complete circumferential resections within the allotted time.

### Efficiency and speed

Use of the robotic platform reduced the procedure duration. The mean ESD procedure time was 14.1 ± 4.3 min with robotic assistance, compared to 21.6 ± 7.6 min with the conventional technique. This corresponds to a ~ 35% reduction in resection time (*P* = 0.028). In fact, all operators completed their robotic ESD cases comparatively faster than their conventional cases. This improvement in efficiency was also reflected in the dissection speed. The robotic system enabled a higher dissection speed of 36.5 ± 23.4 mm^2^/min, more than double the 16.3 ± 10.1 mm^2^/min achieved with conventional ESD (*P* = 0.05). Notably, within-operator comparisons showed that all four participants, regardless of their prior experience level or the order of modality, achieved faster procedure times in their robotic cases compared to their conventional cases.

### Tissue injury and safety

No full-thickness perforation occurred in any of the 16 cases (robotic or conventional). However, injury to the muscularis propria was observed in the majority of the conventional ESD cases. Specifically, 5 out of 8 conventional cases (62.5%) had evidence of inadvertent muscularis damage (*n* = 4 grade 1 muscularis propria exposure, *n* = 1 grade 2 torn muscularis propria), compared to 0/8 robotic cases with any muscle injury. This suggests a trend toward improved safety with the robotic system, though the difference did not reach statistical significance (*P* = 0.20) due to the small sample.

### Operator workload and ergonomics

There was a reduction in perceived operator workload and perceived strain when performing ESD with the robotic platform. The median NASA-TLX score (0–100 scale) across all domains was lower in the robotic group compared to the conventional group. In particular, the robotic system minimized the physical effort required (i.e., instrument motions were performed via finger movements on the pen controller rather than large wrist motions on the endoscope) and reduced frustration levels, as the second instrument allowed better control. Subjectively, all four operators reported that the robotic ESD felt “less strenuous” and that they experienced far less fatigue in their hands, wrists, and shoulders compared to doing the conventional cases. They could remain seated comfortably, as opposed to contorting their posture to manipulate a traditional endoscope. Similarly, NASA-TLX sub-domain scores for mental and temporal demand, effort, and frustration ratings were lower for robotic ESD (Table 2).

**Table 2:** Summary of subjective ergonomic assessment between the robotic versus conventional endoscopic submucosal dissection: National Aeronautics and Space Administration Task Load Index (NASA-TLX), Objective Structured Assessment of Technical Skills (OSATS), and Global Evaluative Assessment of Robotic Skills (GEARS)

		Robotic (*n* = 8)	Conventional (*n* = 8)	*P*
NASA-TLX score				
	Mental Demand	46.3 (29.5)	78.1 (12.5)	0.024
	*Physical Demand	26.9 (22.8)	76.3 (9.5)	0.0007
	*Temporal Demand	35.6 (25.7)	71.9 (17.1)	0.011
	Performance^+^	23.8 (23.9)	67.4 (26.5)	0.009
	*Effort	43.1 (25.8)	81.3 (10.9)	0.008
	Frustration	31.3 (29.9)	75.0 (23.9)	0.004
OSATS score				
	Respect for Tissue	3.7 (0.7)	3.6 (0.7)	0.60
	*Time and motion	3.3 (1.0)	3.3 (1.0)	0.82
	*Instrument handling	3.6 (0.9)	3.8 (0.9)	0.35
	*Flow of operation	3.4 (0.8)	3.0 (1.2)	0.2
	Knowledge of instrument	3.8 (0.7)	3.8 (1.4)	1
	*Use of assistants	3.0 (0.5)	3.5 (0.5)	0.001
	Overall performance	3.4 (0.9)	3.4 (1.1)	1
		1st Robotic (*n* = 4)	2nd Robotic (*n* = 4)	*P*
GEARS score				
	Depth perception	3.5 (0.8)	3.4 (0.9)	0.60
	*Bimanual dexterity	3.5 (0.5)	3.6 (0.7)	0.60
	*Efficiency	3.1 (1.2)	3.6 (1.1)	0.28
	Force sensitivity	3.4 (0.7)	3.6 (0.9)	0.35
	*Robotic control/autonomy	3.5 (0.8)	3.9 (1.1)	0.20

^*^Ergonomic domains, continuous variables (mean, SD)

^+^Performance item is reverse-scored, lower scores indicate greater perceived task success

In terms of technical skill assessments, the OSATS scores were similar between robotic and conventional ESD in most domains, reflecting that the overall performance was adequate in both. However, one notable difference was in the “use-of-assistants” domain: in conventional ESD, the fellows frequently needed an assistant (i.e., help in exchanging instruments or injection), whereas in robotic ESD, the built-in second arm obviated this need. Consequently, the OSATS use-of-assistants metric was better (lower reliance) in the robotic group. The GEARS evaluations for the robotic cases suggested fast adaptation to the system and there was a trend of improved efficiency and bimanual dexterity scores in the second robotic case compared to the first for each operator, though differences were not statistically significant given the small sample. Outcomes stratified by individual operator and technique are presented in Supplementary Table 1.

## Discussion

In this preclinical study, a novel dual-arm endoluminal robotic platform was associated with improved ESD performance in this pilot model. Compared with conventional ESD, robotic assistance resulted in a trend toward faster resection and lower operator workload while improving procedural safety. These findings support the premise that surgical-style triangulation and ergonomic control can meaningfully address technical difficulties and operator fatigue that hinder broader ESD adoption. Importantly, even with only minimal training on the robotic system, novice and intermediate operators achieved en bloc resection in all cases using the robot, which is not always possible with the conventional method.

The expanding literature on robotic-assisted endoscopy encompasses a heterogeneous group of technologies that differ substantially in design, capability, and clinical intent. Existing systems can broadly be categorized as modular single-arm robotic attachments that provide traction assistance through conventional endoscopes (e.g., RoboPera^™^, EndoRobotics, South Korea) or fully integrated standalone robotic systems (e.g., EndoDrive^™^, EndoQuest Robotics, USA or EASE^™^, Endomaster, Hong Kong) [11, 12, 19, 20]. To date, however, no prior study has specifically evaluated a robotic platform that enables true bimanual manipulation—simultaneous independent traction and dissection—while remaining fully compatible with standard therapeutic gastroscopes used in routine clinical practice. This distinction is important, as bimanual control represents a fundamental technical advance over traction-only assistance and more closely mirrors the principles of surgical triangulation that underpin efficient and safe dissection. Delivering this capability without requiring a proprietary endoscope or major workflow redesign has important implications for translational feasibility and scalability. In addition, the portable positioning cart permits disengagement of the robotic arms while leaving the endoscope in situ, allowing seamless reversion to conventional ESD without scope exchange. Importantly, because traction and dissection are controlled directly by the primary operator, the system obviates the need for a second highly trained assistant to manipulate a robotic traction device—an approach required by some existing endorobotic platforms and one that demands substantial expertise in ESD planes and technique.

Within this context, the present study builds upon prior preclinical and early clinical investigations on robotic-assisted ESD. In an ex vivo randomized study, de Moura et al. showed that ESD-naïve endoscopists achieved a 100% en bloc resection rate using a dual-arm robotic system versus 50% with conventional ESD, alongside a > 60% reduction in procedure time and significantly lower physical and mental workload scores [8]. Similarly, other ex vivo and in vivo porcine studies have shown that robotic ESD increases dissection speed, reduces blind cutting, and minimizes muscular injury compared with standard techniques [10, 12]. Our findings build on this body of work: robotic assistance resulted in faster resections, universal en bloc outcomes, and markedly improved tissue preservation even among relatively inexperienced operators who were only given 30 min to complete the task. Notably, no robotic cases in our study incurred muscular injury, whereas more than half of conventional cases did. This mirrors trends reported by de Moura et al., who also observed fewer perforations with robotic assistance [8]. Although our study was underpowered to demonstrate statistical significance, these numerical differences are clinically meaningful—a 25% absolute improvement in en bloc resection affects curative intent, and complete elimination of muscular injury reduces the risk of post-ESD electrocoagulation syndrome, underscoring the need for larger prospective in vivo studies to confirm these trends. Operators consistently found it easier to maintain the correct submucosal plane when traction and dissection could be performed simultaneously and independently. During difficult portions of conventional ESD, by contrast, tissue would occasionally slip or tent unpredictably, increasing the likelihood of inadvertent deeper cuts. This qualitative feedback provides important context for the observed quantitative differences in tissue injury and highlights how bimanual manipulation may be advantageous for maintaining safe dissection planes.

Early clinical experience with robotic-assisted endoscopy further supports these observations. A recent pilot randomized trial (*n* = 48) in patients with early gastric neoplasia demonstrated that a single-arm traction robot significantly reduced muscular injuries during gastric ESD compared with the conventional technique, despite similar procedure times and uniformly high en bloc and R0 resection rates in both groups [7]. Likewise, the first-in-human trial (*n* = 43) of a fully integrated endoscopic robotic system for colorectal ESD reported high *en bloc* resection rates (94.6%) with acceptable procedure times (median 49 min) and a low incidence of adverse events (2/43 patients) [21]. Although the type of endorobotic system is different, these studies confirm that the technical advantages observed in laboratory settings can translate into clinically meaningful benefits.

Conventional ESD is not only technically demanding but also physically taxing; repetitive strain, awkward postures, and sustained forceful maneuvers contribute to a high prevalence of work-related musculoskeletal injury among endoscopists, reported in 30–89% of practitioners [22]. In our study, robotic assistance reduced perceived physical and mental workload. By allowing operators to work seated, with minimal force and intuitive hand movements, and by offloading traction to a dedicated robotic arm, the platform mitigated many of the ergonomic stressors inherent to conventional ESD. Notably, these perceived benefits were realized after only brief familiarization, suggesting that the learning curve for the robotic interface itself is short. Even endoscopists with no prior robotic surgery experience became proficient after a short practice session and a single trial case. These findings have implications for training and dissemination. By mitigating technical and ergonomic barriers, robotic assistance may lower the threshold for technical proficiency and expand access to ESD beyond a small number of high-volume expert centers. If robotic platforms can reduce physical strain while simplifying key technical steps, trainees may be able to perform more cases with less fatigue, potentially accelerating skill acquisition, while experienced endoscopists may be better able to sustain long-term practice. If robotic assistance is shown to reduce the number of cases required to achieve technical competence, this technology could substantially alter current ESD training paradigms. In parallel, ongoing engineering development is focused on expanding instrument capabilities, including needle injection and suturing through the robotic arms, as well as the integration of machine vision or augmented guidance to assist with submucosal plane identification and anatomic orientation.

This study should be interpreted considering these limitations. First, it was conducted in an ex vivo porcine stomach model, which cannot perfectly replicate the clinical scenario. While using explanted tissue allowed us to control lesion uniformity and eliminate patient-related variability, in vivo studies will be required to evaluate system performance under physiologic conditions, particularly with respect to platform stability and instrument control when the endoscope is parked at a target lesion during active peristalsis, respiration, and bleeding [23, 24]. As such, the observed technical and ergonomic benefits should be viewed as hypothesis-generating rather than definitive evidence of clinical superiority. Second, the sample size was relatively small, which limits the statistical power for some endpoints (i.e., en bloc resection and muscle injury rates did not reach significance despite clear numerical differences). A larger series would better delineate the consistency of these benefits and potentially reveal significant differences in those outcomes. Third, the operators in this study, while not experts in ESD, were trained endoscopists (fellows) with familiarity in general and advanced endoscopy. On the other hand, we did include varying experience levels, and both groups seemed to benefit from the robot. Our reliance on subjective metrics, specifically the ergonomic outcomes and NASA-TLX workload survey and unblinded visual assessment of muscular injury, introduces the potential for measurement and novelty bias. These assessments should be considered exploratory rather than definitive. While the presence of two independent expert observers was intended to minimize individual bias, the inherent enthusiasm for a novel robotic platform may have influenced perceived effort scores. Future studies should employ blinded video review by independent raters. Another limitation is that we did not formally measure the setup time required for the robotic platform or compare the overall procedure workflow (e.g., time for device deployment, etc.). In practice, the few extra minutes needed to mount the system on the scope could be a consideration, though the formal setup and total workflow time were not measured. Fourth, the lack of standardization between the energy platforms serves as a technical confounder. Dissection speed and tissue injury rates may have been influenced by the inherent properties of bipolar versus monopolar energy rather than the robotic platform alone. However, since the ex vivo nature of the tissue eliminates the variable of bleeding, the observed benefits in procedure efficiency and triangulation are more likely attributable to the bimanual robotic control than the energy modality itself. Finally, while our results on ergonomic improvement are compelling, they rely on subjective metrics (i.e., surveys and ratings). Objective measurement of operator posture or muscle load such as motion tracking or electromyography could further substantiate the ergonomic advantages in future studies.

## Conclusion

The present pilot study demonstrates that introducing surgical triangulation and bimanual dexterity into flexible endoscopy through a novel dual-arm robotic platform compatible with standard endoscopes can potentially enhance the efficiency, safety, and ergonomics of ESD. These improvements can potentially address two major barriers that have limited ESD adoption: technical complexity associated with a prolonged learning curve and the substantial physical demands placed on the operator. These preclinical study findings suggest that a dual-arm robotic platform may address technical and ergonomic barriers; however, given the small sample and the influence of early-phase learning, these results should be viewed as hypothesis-generating support for future longitudinal in vivo studies. With continued refinement and clinical validation, such robotic platforms may represent an important step toward a new paradigm in therapeutic endoscopy, enabling complex interventions to be performed with greater precision, consistency, and operator sustainability while maintaining a minimally invasive approach.

## Supplementary Information

Below is the link to the electronic supplementary material.

Supplementary file1 (XLSX 11 KB)

## Abbreviations

ERBE: Elektromedizin GmbH
ERBEVIO300D: ERBE VIO^®^ 300 D electrosurgery unit
ESD: Endoscopic submucosal dissection
FDA: U.S. Food and drug administration
GEARS: Global evaluative assessment of robotic skills
IACUC: Institutional animal care and use committee
NASA-TLX: National aeronautics and space administration task load index
NOTES: Natural orifice transluminal endoscopic surgery
OSATS: Objective structured assessment of technical skills
SAR: Special administrative region
SD: Standard deviation
